# Decolonisation through Digitalisation? African Languages at South African Universities

**DOI:** 10.1007/s41297-023-00196-w

**Published:** 2023-05-09

**Authors:** Irina Turner

**Affiliations:** Afrikanistik I, University of Bayreuth, 95440 Bayreuth, Germany

**Keywords:** isiXhosa, Translingualism, Digitalisation, Intellectualisation

## Abstract

Many South African universities aim to implement one or more African languages into formal and digital structures, in a process known as localisation. Often, however, this objective is not met due to manifold obstacles, not least of which is a conceptual contradiction of bounded languages and translingual practices. Digitalisation promises to diversify teaching and learning experiences as well as knowledge production at universities. At the same time, it threatens to reproduce analogue power structures. How do institutions negotiate these potentials and pitfalls? This paper explores the digitalisation of isiXhosa at three South African universities in the Eastern Cape province: Nelson Mandela University, Rhodes University, and the University of Fort Hare. Qualitative methods such as discourse analysis and immersed observation were applied to describe state-of-the-art developments in the digital implementation of isiXhosa in the realms of outward representation and communication, learning spaces, and knowledge repositories.

## Introduction

In 2019, language experts gathered in Makhanda, South Africa, for a workshop aimed at increasing awareness of the South African language isiXhosa. The United Nations declared 2019 the Year of Indigenous Languages. Andiswa Bukula, representative of the South African Centre for Digital Language Resources (SADiLaR), stressed the importance of building digital capacity for African languages to promote their status, prestige, and use in formal spaces. A major stumbling block in the digital development of African languages has been the comparatively meagre availability of digital text corpora that enable algorithms to build useful tools and resources for education (Roux, 2020). SADiLaR, established in 2019 (Ventures Africa, 2019), produces and maintains freely available digital language annotated corpora in all South African languages; today, it hosts 96 resources in and for isiXhosa (SADiLaR, 2022).

This is positive news with regard to decolonising university space that, on a theoretical level, implies linguistic transformation and inclusion of African languages as a central concern (Wa Thiong’o, 2011[1986]). Decolonial transformation can potentially gain a lot from digital transformation because they both plurify normative ideas of knowledge production.

In terms of—theoretically available—technological infrastructure enabling multilingualism, South Africa seems to be making big leaps in some areas, such as literacy development (Durodolu & Mojapelo, 2020), but moving slowly but steadily in others such as, for instance, providing laptops for studying during Covid-19 lockdowns (Macupe, 2021). Gross inequality in access to digital infrastructure and digital literacy education remain the biggest challenges (Hanekom, 2020; Matli & Ngoepe, 2020). Advances in these areas would, according to Don Osborn (2010), automatically boost the digital presence of African languages.

Potentially seen as the epitome of indigenous knowledge, languages contain not only semantic concepts describing life worlds but also ontologies that relate to holistic spiritual and philosophical existence. In the process of digitally decolonising universities, integrating African languages and translingual practices is a crucial condition for social and epistemic justice. To what extent has this integration been realised at South African universities? Starting from the assumption that higher education drives intellectual transformation, this article explores the advancements of digitalisation at three South African universities where isiXhosa is a language spoken by the majority but not fully implemented in the institutions.

After explaining the conceptual grid of decolonisation, the language question, and digitalisation, the article gives an account of methods and ethical considerations. The study draws on an array of qualitative research methods such as immersed observation, linguistic landscaping, and discourse analysis. The subsequent analysis examines digital isiXhosa in several fields at three South African universities in the Eastern Cape, a province where isiXhosa is spoken by most of the population. The results are discussed in the tension field of standardisation versus a multiplicity of communicative practices. To conclude, I stress the need for an officially acknowledged and fostered translingual approach.

## Decolonisation, digitalisation, and the language question

The study is temporally, locally, and epistemically situated in the context of decolonisation in higher education (Woldegiorgis et al., 2020), which converges with digital transformation. It explores how, in three universities in South Africa’s Eastern Cape province, the so-called language question (Alexander, 2012) plays out in this tension field between the normalising blueprint of digitalisation and the pluralistic agenda of decolonisation.

### Coloniality in higher education

Decolonisation is concerned with productively and justly redressing colonial structures evident in the continuing dominance, imposition, and normativity of Western scientific paradigms, practices, and provenance of people. For the study of the South African education system, decolonial thinking offers ideal frameworks and concepts for a deeper understanding of post-apartheid South Africa’s intricate social complicities and the persisting historically founded “racial geography” of inequality (Christie, 2020, p. 4). Epistemic unsettling is the common ground within the decolonial impetus. Coloniality—first mentioned by Aníbal Quijano (1993)—denotes the ongoing, entangled, unresolved effects and maintained structures of cultural and epistemic colonial violence (Ndlovu-Gatsheni, 2013). Furthermore, it has been argued that the university’s production of standardised knowledge and fostering of resource-dependent competition can be seen as the epitome of coloniality (Mbembe, 2016; Santos, 2017; Swartz et al., 2019).

A prime example of epistemic coloniality is linguistics. Communicative practices are one of the most evident manifestations of unequal power structures, which is why the treatment of languages in educational institutions is central to the decolonial project.

It is generally problematic to speak of ‘African languages’ due to their provenance in colonialism and ongoing negative effects into contemporary social structures, and not only in South Africa. African languages have been “invented” (Makoni & Mashiri, 2007) by colonisers who put perceived linguistic idiosyncrasies and semantics into grammar constructs based on Western models (Gilmour, 2006). The linguistic study of “indigenous” African languages was part of a colonial project that violently ostracised and “othered” Africans (Makoni & Meinhof, 2004). The contemporary translingual turn in linguistics research is conscious of this heritage and acknowledges the ubiquitous hybridity of communicative practices beyond written standards (e.g. Makoni & Pennycook, 2012). Within this consequential thinking, there is now an attempt at avoiding language labels or even the term “languages.” Yet, in writing about “the language question” at universities, as this text attempts to do, re-using these terms and labels and describing the conceptual structures of the institutions is unavoidable.

### The language question

An unresolved riddle in post-apartheid South Africa, the so-called language question (Alexander, 2012), refers to the presence and role of African languages beside English and Afrikaans in higher education. This topic has been discussed by scholars and practitioners for many decades (e.g. Cloete & Maassen, 2002; Thamaga-Chitja & Mbatha, 2012; Webb, 1996; Wolff, 2017). The language question has been prevalent since the “invention” of African languages but remains unresolved due to its complex alignments with identity and power structures (e.g. Makoni & Mashiri, 2007; Zeleza, 2006).

Strikingly, the #FeesMustFall discourses of 2015/2016, for instance, which called for decolonisation of the university space, did not feature African languages as a primary objective (Luckett & Hurst-Harosh, 2021). This is partly due to the historically grounded baggage of African languages as colonial constructs (Gilmour, 2006), which puts home language speakers of non-colonial languages in an uncomfortable and seemingly unresolvable predicament: asserting African identity by reiterating colonially grounded categories or resorting to English in the hope for social and economic equity (Rudwick, 2021). The language question is, nevertheless, not a side-line aspect of social justice, because “the way languages are constructed has an impact on the material circumstances of Africans” (Makoni & Mashiri, 2007, p. 81). Therefore, in the most unequal country in the world (The World Bank, 2022), this is a crucial issue stretching far beyond the university walls. However, institutionalising translingual practices (e.g. Hibbert & van der Walt, 2014) seems like one solution that requires fundamental systemic changes in terms of processes and ideologies.

The question underlying the language issue in tertiary education is how an institution’s stabilising mandate—providing reliable and operational knowledge—can be harmonised with epistemic diversity. On the one hand, linguistic forms and rigid boundaries need to be disrupted, while on the other hand, semiotic cohesion needs to be emphasised (Makalela, 2021). Conscious hybridity might be one solution, but it also requires institutional acknowledgment and utilisation of multiplicity for normative purposes to achieve social justice. Linguistic decolonisation is therefore a process far more complex and ambiguous than simply replacing one language with another.

In the case of asserting isiXhosa in formal spaces and through the vehicle of English, the transformation has been termed Xhosalising (Paxton & Tyam, 2010). Going beyond language replacement, code-switching, or semantic reappropriation, Xhosalising is an epistemic infiltration of cultural and social domains dominated by English. Instead of “isiXhosa being Anglicised … English is being Xhosalised”, i.e. strategically modified towards a newly negotiated meaning for Xhosa-sensitive contexts of communication and identity (Paxton & Tyam, 2010, p. 255). Xhosalising exceeds grammar by incorporating deeper semiotic/cultural indices such as images, sounds, gestures, metaphors, and style.

Zhou and Landa (2019) find that in stressing the English/isiXhosa binary, Xhosalising falls short of representing translingual practices and multilingual realities at the university, even where isiXhosa is a majority language. The authors thus argue for a consequent translingual approach, also on policy level, in South African higher education (Zhou & Landa, 2019).

Yet certain African languages, such as isiXhosa, is still being strategically “developed” under the framework of so-called intellectualisation that aims to foster the learning, employment, and appreciation of African languages for and in formal and official spaces (Nkomo, 2020). Central concerns of intellectualisation are implementing policy, publishing and researching glossaries, and developing terminology in and of African languages, as well as using information and communication technologies (ICT) to do so (Kaschula & Maseko, 2017). While intellectualisation seems to implicitly suggest an epistemic deficiency of African languages, Kaschula and Maseko (2017, p. 20) assert that it is precisely not about catching up in linguistic complexity but about addressing the material gap concerning African languages caused by “years of neglect and the lack of both corpus and status planning” and thus propose strategic development in higher education teaching and learning.

On a critical note, intellectualisation can, nevertheless, be conceived as a neo-colonial move filling a pre-constructed void linked to the assumedly missing sophistication of isiXhosa speakers. It operates with the same modernistic tools colonisers applied to “domesticate” African languages such as prescription, measurement, and authoritative judgement on inclusion and exclusion. Practically, there are, however, few alternatives to the intellectualisation project if a minority world notion of university, epistemology, and learning are to be harmonised with “indigenous” ways of being and knowing in the world.

### Digitalisation

As part of intellectualisation, digitalisation can play a role in the dissemination and accessibility of information. Digitalisation is the binary coding of information, knowledge, and social practices, and can thus be seen as a type of standardised linguistic translation. Nevertheless, it exceeds the mere creation of a symbolic machine-readable representation from the analogue sign by encompassing the migration and transformation of social structures to digital logic and technology (Innerhofer et al., 2020). It is therefore an epistemic and ontological transformation because what we learn and know through digital means influences what we can conceive.

Digitalising isiXhosa means an increased and diversified online presence for the language. It does not downgrade isiXhosa’s sophistication but rather asserts its status as a fully legitimate South African language in every social realm.

A type of synonym for digital Xhosalisation is localisation, which refers to the adaptation of ICT content and interfaces to “local modes of communication, culture and standards” that can be seen as a digital extension of social structures (Osborn, 2010, p. 7). Language is central to localisation—which, in turn, is subject to the limits and potential of hardware and bits (binary encoding) of computer systems (Osborn, 2010). Updating software and hardware (e.g. fonts but also speech recognition), producing multilingual web content, and creating suitable interfaces are the three main working areas for localisation (Osborn, 2010). Current technology enforces and reinscribes linguistic categorisations and language boundaries and often makes efficient digital representation of varieties and mutually intelligible languages difficult, resulting in some languages being strengthened over others (Osborn, 2010).

## Methods and ethical considerations

The study is anchored in decolonial linguistics and Southern sociology of language, which strives to re-centre the Southern experience, identify suitable Southern theory, interrogate universalist claims in Northern models, and address structural, gendered, and racialised inequality within academia (Rudwick & Makoni, 2021).

The central approach to this enquiry is discourse analysis, i.e. a contextual and critical reading and synthesis of related texts. The corpus is mainly based on a review of literature (policies, case studies, and journal and newspaper articles) referring to the digitalisation of South African languages and isiXhosa in particular. Most of these texts emerged from applied linguistics and educational studies. In selecting these sources, I focused on actuality, i.e. articles from within the last 15 years; region (the Eastern Cape, where isiXhosa is prevalent); and research from scholars based in South Africa. The data used in this article forms part of a larger book project for which about 720 sources were consulted, and several expert interviews conducted (2018–2023).

The analysis is furthermore enriched by digital field grazing, i.e. accessing university websites and available repositories, but also immersed observation on site, e.g. as a guest lecturer, speaker, and conference participant, as well as an approved external researcher between 2015 and 2019. I was able to scrutinise internal digital practices and linguistic landscapes, i.e. observable communicative and semiotically charged physical/digital manifestations of isiXhosa in and around university spaces.

I am aware of my position as an outsider—a white researcher based in Germany—and what this means for my observations. My insights will always lag current developments and milestones in digitalisation at the universities. Furthermore, with my academic background in African linguistics, I risk reiterating power structures of North-South coloniality. I am aware of these limitations yet nevertheless believe that this study could contribute to the body of knowledge by capturing an important moment in the digitalisation of isiXhosa in tertiary education, as well as contextualising its political and social implications.

## Analysis: areas of digital Xhosalisation

Despite their elite status, universities are knowledge laboratories not least on the effects of digitalisation on communication, and they can serve as fruitful sites of enquiry for society at large (Klein et al., 2020). This case study encompasses three universities in the Eastern Cape, a province in South Africa where isiXhosa is spoken by most of the population. At these three tertiary institutions, decolonisation has moved to the forefront of discourses and practices.

### Institutional context

The University of Fort Hare (UFH)—with campuses in East London, Alice, and Bisho—is historically strategically disadvantaged. It is a so-called “Black” university and perhaps best known for its prestigious political alumni including Nelson Mandela (UFH, 2021) as well its history of political resistance (Massey, 2010). It was only in 2013 that UFH relaunched the previously monolingual English policy of 2006 and officially “became” trilingual. In theory, isiXhosa, English, and Afrikaans all have equal status in teaching and administration (UFH, 2018), yet in practice, trilingualism has been implemented very slowly, as my on-site observations and conversations with several staff members confirm, and this despite UFH’s very high proportion of isiXhosa home language speakers.

Nelson Mandela University (NMU), based in Gqeberha, is a so-called comprehensive university that historically has an applied/technical focus (Essop, 2020). In 2018, 50% of students were isiXhosa-speaking (Batyi, 2019). NMU’s language policy dates back to 2009 and takes an “add-on” approach in the sense of bridging a proficiency gap of African language speakers towards English or Afrikaans. It does not explicitly address translingual practices (Coetzee-de Vos, 2019, p. 59). Despite this reluctance to fully endorse multi- or even translingualism, NMU engages in fundamental holistic transformation efforts such as decolonising the linguistics curriculum (e.g. Lueck, 2020) or renaming buildings (NMU, 2019).

Rhodes University (RU), based in Makhanda, is a so-called research intense university (Essop, 2020). A formerly “white” university, it is known as a liberal institution that openly opposed apartheid policies (Bunting, 2002; for a more critical stance, see Hendricks & Vale, 2005). Today, RU staff and students have about 22 different home languages among them, but most name isiXhosa as their home language (Resha, 2019). Even though RU adopted a multilingual language policy in 2005 and revised it in 2014 (Gambushe et al., 2017), it still clearly favours English over isiXhosa, which is seen as holding a lower intellectualisation status. The policy loosely pledges to assist bilingual communication in situations where English proficiency is lacking (RU, 2014) and thus reiterates existing power structures. RU—despite efforts to boost Xhosa language and identity—remains an “English university”, as its students and staff favour this language in learning, teaching, and research (Resha, 2019, p. 8ff).

Each of the three universities has its own historically founded idiosyncrasies, which means each institution has its own resources, baggage, and identity and ultimately Xhosalises in its own way. Nevertheless, in principle, all three face the same obstacle of finding ways to de-anglicise the institution and boost isiXhosa and other African languages.

### External and internal communication

A website is the face of an institution, and reflects its structure, values, and activities. It is seldom interactive and rather a digital promotional brochure and reveals the institution’ cultural identity, whether it be authentic or aspirational.

Within the framework of intellectualising isiXhosa, NMU’s aim to provide an isiXhosa website by 2015 (NMU, 2018) had not been realised by 2022, when this research was conducted. It only had an English website and language could not be switched. Similarly, UFH offered only a few subpages of its website in isiXhosa. The only website that provides direct translation of all pages—via a Google plug-in—is that of RU.

The absence of African languages in official external communication defines an institutional in-group (Scollon & Scollon, 2004), of English speakers, and reveals that multilingualism remains a theoretical concept in the digital realm.

Some official email communication with staff, however, takes place in isiXhosa. For example, in NMU’s 2021 staff well-being survey, participants could answer in three languages (English, Afrikaans, isiXhosa)

While an official website statically represents an institution and is preferred as an information archive, social media engagement on an institutional level is much more dynamic and thus increasingly important for public visibility, representation, communication, and institutional identity. Broadly defined, social media are web-based one-to-many communications; information shared on social media typically requires low-threshold access (Bosch, 2017). Within these networks, groups of common values emerge, and users’ active (dis-)alignment from or with these groups, through sharing and commenting, is a vital part of virtual identity work.

All three universities have Facebook, LinkedIn, Twitter, and YouTube pages; UFH and NMU also each maintain an institutional Instagram account. Except for a few visitor comments, all official entries on these institutional pages are in English.

Social media opens a space for discourse and, of course, runs the risk of counter-discourse, confidentiality leaks, so-called shitstorms, and other breaches. The RU renaming issue in the aftermath of #FeesMustFall 2016, for instance, resulted in the student representative council creating an alternative social media space to the official institutional one (Resha, 2019). Translingual African language social media posts associated with institutions of higher learning—where formal and informal representation intersect—foster semiotic visibility and increase the value of these languages.

Furthermore, I noticed that among each other, but also in communication with lecturers, students use social media such as WhatsApp to communicate, often in isiXhosa and in translingual registers. Unique conventionalised social media abbreviations and shortcuts are employed, in social conversations in particular. Especially during lockdown, university-enabled social media became the preferred space to negotiate identity related to being or becoming a university student (DeAndrea et al., 2012). Social media, Babalwa Resha (2019, p. 42) argues, reflect the dominant culture “to which African youth bring a unique reality of their worldview”*.* Thus, social media hold great potential for the momentum of decolonisation.

### Learning and teaching

Covid-19 accelerated the use of social media in South African classrooms (Motala & Menon, 2020) and broadened spaces for Africanised communication. For instance, Leketi Makalela (2021, pp. 11ff) reports how comfortably students use Facebook in class to produce translingual “African language [Sepedi] content”. However, there seems to be little concern about the potential risks posed by these commercially oriented tools in formal teaching. Social media are easy to use, widely available, and almost free of charge. Yet students and staff pay with information on their identity. Open social media put sensitive and valuable data, as well as intellectual property, at risk. Monitored, exclusive e-spaces are better suited to academic communication and learning.

RU uses the predominantly English platform RUconnected for students’ self-management, interactive communication, archiving, and provision of study content. In 2019, when I conducted the research for this article, RUconnected already implemented Afrikaans but did not yet have an isiXhosa interface. UFH’s Blackboard also had no isiXhosa options in 2021, and neither did NMU’s Moodle. A culture-sensitive and semiotically broad e-learning environment—including icons, pictures, and symbols—is to a large extent still a desideratum in South Africa (Chukwuere et al., 2016).

In 2016, NMU experimented with multilingual digital learning spaces. During a tutorial, tourism students were encouraged to “code-mesh” (English/isiXhosa) and exchange in a more informal digital environment where their cultural and linguistic backgrounds were appreciated (Batyi, 2016). The students needed a great deal of encouragement to unlearn mono-lingual conventions and expectations and to slowly start trusting the digital space as safe for identity expression (Batyi, 2016).

The 2016 NMU experiment seems to show that, while digitalisation can enable decolonial learning, traditional hierarchical structures, power distribution, and conventional teaching methods are not automatically removed from the virtual realm. In other words, technology does not democratise and bring about a shift in ideology per se. Online teaching is often still driven and controlled by conventional gatekeeping and power structures, further ensuring the hegemony of English.

For many young South Africans, a potential asynchronous, cost-effective alternative to face-to-face tuition is the often-free massive open online course (MOOC) developed and provided by universities as part of their politically encouraged digitalisation agenda and third mission strategy. Even though most of these courses are only available in English, their accessibility and flexible structure display aspects crucial for decolonial education and life-long learning (Hone & El Said, 2016). While the three Eastern Cape universities included in this study might have started to develop MOOCs, they are certainly not yet broadly advertised and cannot easily be found on the standard platforms (MOOC List, 2022).

Generally, the perceived impediment to prioritise on MOOCs might also be due to the institutions lacking resources. Furthermore, the added value of MOOCs is contested as they are sometimes perceived to be of low quality and often aim to promote full access to paid content (Fatumo & Adelabu, 2020). On average, fewer than 10% of MOOCs are completed (Hone & El Said, 2016), which suggests that digital distance learning is not always an optimal tool. This applies in particular to the large proportion of South African students who live and study in environments that are neither conducive to concentration nor guarantee affordable and reliable connectivity.

Ahmed Essop (2020) thus recommends a mixed approach with some distance learning and some contact teaching. In the aftermath of the Covid-19 pandemic, universities were encouraged to prioritise blended learning (Motala & Menon, 2020), the integration of online tools and resources with face-to-face teaching and studying. UFH, for instance, used social media like WhatsApp for online teaching and digitalised study material, making it available on their learning platform (Linden, 2020). Digital offers can be individualised offering potential for translingual approaches, e.g. through multilingual chats, automated translation, asynchronous learning, and need-based automated repetition.

However, as initial observations suggest, offline linguistic power structures are mirrored in blended-learning spaces not least due to the monolingual orientation of lecturers (Coetzee-de Vos, 2019). While at first sight, online-only and blended environments sound compatible to decolonial learning in multilingual settings, digitalisation is not per se increasing epistemic justice.

Digital intellectualisation in tertiary education has enabled the establishment of course-related multilingual glossaries and dictionaries. Increasingly, these texts are conceived of as nascent digital products instead of merely being “PDF-ed”. Instead of copying paper products, electronic resources should be “born digital” to use all features available through digitalisation (e.g. hyperlinks and phonetic audio-representations) (Prinsloo & Zondi, 2020). Digi products should generate meta data and individualised support to guide users through the grammatical complexities and structural orientation in situ, i.e. while using the dictionary. These dictionaries should be linked to big electronic corpora (e.g. at SADiLaR) (Prinsloo & Zondi, 2020).

Current projects try and avoid simply copying analogue resources and dictionary structures, by integrating frequency scales, clustering themes or semantics, or through word embedding computing based on semantic or syntactical similarity (Eckart et al., 2020, p. 10ff). Several undertakings, such as African Wordnet (Griesel & Bosch, 2020), make existing digital language data sets—such as dictionaries—openly available to users ranging from primary school pupils to researchers.

With regard to digital language repositories, UFH, NMU, and RU have built on their analogue efforts with first language speakers developing isiXhosa and multilingual glossaries to counter the argument that African languages are limited in higher learning due to the lack of expert terms.

By 2019, NMU had 25 multilingual digital glossaries on Moodle (Batyi, 2019) as well as on the university website. Today, there are many subject and course-specific glossaries and dictionaries in African languages at several South African universities (e.g. Madiba, 2014).

In 2021, RU joined an international initiative called BAQONDE that promotes African languages at the university and language policy implementation (RU, 2021). A core focus of the project will be digitising multilingual and multimodal material, such as recordings of lectures with subtitles, annotated key readings, and glossaries (Dion Nkomo in RU, 2021).

Some have noted student resistance to multilingual glossaries based on the fear of losing English competencies; non-English speakers also worried about stigmatisation when openly making use of glossaries (Dalvit, 2010). The already mentioned absence of the language debate from #FeesMustFall discourse could be an indication that this stigma lingers.

### Knowledge repositories

Archives are meaningful for contemporary engagement and learning, as well as for projecting knowledges that will be relevant for the future. All three Eastern Cape universities host intriguing digital repositories that do not relate exclusively to isiXhosa as a language but—even more so—to amaXhosa history, epistemology, and ontology and are therefore crucially linked to Xhosalising the university space.

An important part of South African history is housed at UFH’s Centre for National Heritage and Cultural Studies (NAHECS), which contains physical material about South Africa’s anti-apartheid struggle history and liberation movements such as the African National Congress. It is hoped that through the ongoing digitisation of this African heritage collection, a process that commenced in 2011, the archive will become accessible to the greater public (UFH, 2022). By 2018, the project had digitised about 2 million pages, 82,000 photographs, and more than 3000 objects (Larsen, 2018). By February 2022, its website was still under construction.

Another best-practice example is the famous International Library of African Music (ILAM) at RU. Its collection includes printed literature, sound recordings, field research notes, African instruments, and a digital photo archive (RU, 2022). It also publishes the *Journal of the International Library of African Music*. ILAM strives to be a so-called living archive that benefits the public pro- and interactively (Madiba, 2017). It repatriates music recordings to their communities of origin, organises travelling exhibitions, and creates teaching material for schools and other community engagement activities (Madiba, 2017). The ILAM archive boosts oral literature and indigenous knowledge in African languages and epistemologies.

In addition to institutionalised knowledge on and in African languages, there is also freely available relevant content. As one of the most prominent examples of a democratic, open-access knowledge archive, Wikipedia promotes multilingualism even though it started exclusively in English (Pimienta et al., 2009). On the one hand, a multilingual encyclopaedia could transform minoritised languages into powerful tools, in this way creating an inclusive knowledge society if “speakers take ownership of the future of their language” (Pretorius & Wolff, 2020, p. 244ff).

On the other hand, as Ana Deumert (2014) has observed, most authors of Wikipedia’s isiXhosa encyclopaedia are based outside South Africa and are not part of the Xhosa diaspora but rather learners of the language (see also Dalvit, 2010). This is problematic because authors outside South Africa predominately shape the online representation of Xhosa texts, epistemologies and lifeworlds, possibly sometimes without having first-hand experience and knowledge about amaXhosa identity and, through writing, claim the power to define it. Wikipedia demands a minority world conception of knowledge that postcolonial subjects might not necessarily subscribe to, and thus the use of this platform remains limited for African contexts and contents (Deumert, 2014).

While bottom-up repositories such as Wikipedia, due to their neo-colonial positionality, seem to maintain unequal power relations, increased encyclopaedic presence of African languages on the platform grows significant digital data for the development of language and language technology (Pretorius & Wolff, 2020).

## Discussion

The universities’ efforts described above seem to indicate the need to digitalise isiXhosa, but the project needs lobbying and resources.

Makalela (2021) points out two opposing views that exist regarding the effects of digitalisation on African languages: on the one hand, some fear decay of language standards and orthography, e.g. through social media, and therefore eventual language extinction, while on the other hand, others expect accelerated development through sheer increase in digital presence and data offering opportunities to digitally represent translingual offline practices.

Digitally boosting African languages affects not only communicative habits and cognitive channels but also the standards of the languages themselves. The growing use of mobile phones and social media—e-learning and group chats, emails, and tutor communication and so on—in higher education has entered Xhosalised registers into the formal university realm. About 10 years ago, there were still fears that word limits on short digital messages might impede politeness conventions in isiXhosa (Kaschula & Mostert, 2012) or lead to users eschewing isiXhosa for English due to its relative brevity (Deumert & Masinyana, 2008).

Makalela (in Zuma, 2017) argues that the survival of African registers now heavily depends on their digitalisation and comprehensive tapping into the entire spectrum of communication technologies subject to and based on a transformed understanding of languages and languaging as translingual practices spoken by people rather than delineated, fixed, and standardised repositories defined by so-called experts.

According to Makalela (2021, p. 5), speakers of African languages have so far been “disproportionally” disadvantaged through the “monolingual bias” ingrained in voice recognition and mobile learning technologies. Other interactive features like chats and video calls, as well as audio-oriented outputs such as podcasts or voice messages, potentially create spaces for translingual practices but are yet not fully integrated in educational contexts.

An added complication is that the logic of digitalisation -- i.e, the language of algorithms itself -- necessitates taxonomies, definition of categories, as well as non-ambiguous allocations. In other words, digitalisation needs standardisation, which directly opposes spoken practices and linguistic multiplicity.

Nevertheless, the Internet of Things, i.e. digitally enhanced physical objects of daily life such as interactive speakerphones and monitoring wristwatches, might emphasise the embodied pragmatics of language. The Internet of Things favours practices of languaging and post-linguistic communication emphasising the oral and visual.

Through the increasing online engagement of speakers who also leave written trails of spoken varieties, digital corpora in African communicative practices are growing, which build strategic and relevant non-English corpora as the basis for usable knowledge repositories, e.g. through the work of SADiLaR (2022).

While technical advances increase possibilities for multilingualism and minoritised languages, digitalisation has also sharpened the view on unequal starting positions. When universities shut down during Covid-19 and #FeesMustFall, access as a key indicator of inequality in higher education shifted from formal admission, funding, and system navigation to access to connectivity and digital literacy. Most students struggled to transition to remote digital learning due to cost of data and equipment, as well as absence of conducive learning environments beyond campus—evidence abounds of students and their sponsors having to make a choice “to spend on food or data” (Motala & Menon, 2020, p. 90f).

Digitalisation at universities did not automatically result in an investment in language diversity. Despite making it technically possible, digitalisation does not per se bring about diversification in terms of communicative practices. The hegemony of English prevails, also in the digital realm, and power structures of the analogue world are mirrored online. The presence of delineated African languages in institutionalised online spaces is equally impeded and impeding, as was also observed due to the complexities of the language question. The ambiguities about making use of colonially invented categories, i.e. African languages, for epistemic empowerment continue online.

## Conclusion

This explorative study of the digitalisation of isiXhosa at South African universities provided insights into an ongoing project coinciding with decolonisation and intellectualisation. Besides giving an account of current efforts in digitalisation, the article highlighted connections between these three transformative processes.

Much remains to be done to speed up e-learning and digitally localising African languages in education (Kigotho, 2018), including isiXhosa. The danger in the digital representation of minority languages lies in a tokenistic simplification that reduces these languages to folklore (Grosfoguel, 2007) or exoticises them (Deumert, 2014).

Digitalisation does not necessarily bring about epistemic diversification or social justice unless it is used as a strategic decolonial tool to accelerate the process. Makalela (2021, p. 15) argues that digitalisation of translingual practices can pull down artificial barriers separating African languages and liberate “fluid linguistic identities of their speakers”. In strategising the digitalisation of African languages, it is vital to take a translingual approach right upfront instead of copying offline power structures and normative taxonomies of coloniality.
